# Effectiveness of the blended care self-management program “Partner in Balance” for early-stage dementia caregivers: study protocol for a randomized controlled trial

**DOI:** 10.1186/s13063-016-1351-z

**Published:** 2016-05-04

**Authors:** Lizzy M. M. Boots, Marjolein E. de Vugt, Gertrudis I. J. M. Kempen, Frans R. J. Verhey

**Affiliations:** Department of Psychiatry and Neuropsychology and Alzheimer Center Limburg, School for Mental Health and Neuroscience, Maastricht University, PO Box 616, 6200 MD Maastricht, The Netherlands; Department of Health Services Research and CAPHRI School for Public Health and Primary Care, Maastricht University, PO Box 616, 6200 MD Maastricht, The Netherlands

**Keywords:** Family, Online intervention, Timely, Web-based, RCT

## Abstract

**Background:**

The benefits of e-health support for dementia caregivers are becoming increasingly recognized. Reaching early-stage dementia caregivers could prevent high levels of burden and psychological problems in them in the later stages of dementia. An iterative step-wise approach was employed to develop the blended care self-management program “Partner in Balance” for early-stage dementia caregivers. The design of a study evaluating the process characteristics and effects is presented.

**Methods/design:**

A mixed-method, single-blind, randomized controlled trial with 80 family caregivers of community-dwelling people with (very) mild dementia will be conducted. Participants will be randomly assigned to either the 8-week blended care self-management program “Partner in Balance” or a waiting-list control group. Data will be collected pre intervention and post intervention and at 3-, 6- and 12-month follow-ups. Semi-structured interviews will be conducted post intervention. A process evaluation will investigate the internal and external validity of the intervention. Primary outcomes will include self-efficacy and symptoms of depression. Secondary outcomes will include goal attainment, mastery, psychological complaints (feelings of anxiety and perceived stress), and quality of life. Possible modifying variables such as caregiver characteristics (quality of the relationship, neurotic personality) and interventional aspects (coach) on the intervention effect will also be evaluated. A cost-consequence analysis will describe the costs and health outcomes.

**Discussion:**

We expect to find a significant increase in self-efficacy, goal attainment and quality of life and lower levels of psychological complaints (depression, anxiety and stress) in the intervention group, compared with the control group. If such effects are found, the program could provide accessible care to future generations of early-stage dementia caregivers and increase dementia care efficiency.

**Trial registration:**

Dutch trial register NTR4748.

**Electronic supplementary material:**

The online version of this article (doi:10.1186/s13063-016-1351-z) contains supplementary material, which is available to authorized users.

## Background

Family caregivers are currently becoming the main source of care for people with dementia [1]. However, dementia caregivers are at risk for depression, anxiety and other health problems [2]. Interventions aimed at reducing their burden of care might reduce dementia care costs in the long term if institutionalization can be postponed [3].

Many face-to-face caregiver support interventions have proven to be effective on caregiver distress (random effect size = 0.3), caregiver knowledge (random effect size = 0.5) [4], and self-efficacy (effect sizes ranging from 0.3 to 0.9) [5], but the expected future increase in the number of people with dementia raises concerns about whether care professionals can cope with this demand [6]. E-health interventions could serve as cost-effective alternatives for dementia caregiver support [7–9], both increasing caregivers’ access to support and extending the reach of such support [10–13]. The benefits of e-health are becoming increasingly recognized, and remote support for dementia caregivers is growing [14–16].

Previous e-health studies on dementia caregivers show positive effects on caregiver confidence, stress, depression, and self-efficacy with effect sizes ranging from 0.2 to 0.3 [17]. Provision of tailored information, a coach, and the opportunity to interact with other caregivers shows the most promise [17]. Blending face-to-face guidance with online modules increases caregivers’ connection with the therapist and adherence [18, 19]. However, research on the effects of e-health on dementia caregivers lacks methodological rigor [17], and adverse effects of caregiving are often addressed in the later stages of dementia, when caregivers already feel overburdened [20]. Despite their low levels of life satisfaction and high levels of overload, caregivers do not use services in the early stages because they do not feel the need for such services or because they struggle with acceptance due their experience of stigma [20–22]. Supporting caregivers in the early stages of dementia could prevent high levels of burden and psychological problems in them in the later stages and delay institutionalization [23–26]. However, early-stage dementia caregiver support can be experienced adversely if the care does not suit the caregiver’s personal situation or the stage of the disease. Negative and stigmatizing information can make it difficult for caregivers to identify with and may hamper acceptance [20]. Learning to positively manage life with dementia, instead of managing the dementia itself in a self-management program, could facilitate caregivers’ adaptation to their new caregiving role. A focus on enhancing positive, intact experiences that are tailored to the individual caregiver’s situation may be more effective in increasing caregiver self-efficacy and reducing the negative consequences of caregiving at the later stages [20].

### Study aim and hypotheses

The current paper presents the design of a randomized waiting-list controlled trial investigating the effects of the blended care self-management program “Partner in Balance.” Alongside the effectiveness study, a process evaluation will be conducted to evaluate internal and external validity. As recommended by the Medical Research Council (MRC) Framework for the design and evaluation of complex intervention [27], the program was based on existing literature [17], theory, and user and professional input [28]. In a pilot evaluation, caregivers reported increased feelings of self-efficacy and goal attainment post intervention. Feedback from the feasibility study was used to adapt the intervention to increase user-friendliness [28]. The next objective is to evaluate the process characteristics, effectiveness and cost-consequence of “Partner in Balance.” Specifically, we aim to investigate (1) the internal and external validity of the intervention based on sampling quality (recruitment, randomization and reach) and intervention quality (relevance, feasibility, and performance according to protocol) prior to the effect analysis to evaluate credibility and generalizability [29], (2) whether “Partner in Balance” is superior to the waiting-list control condition in terms of participants’ subjective well-being, as evidenced by improved subjective self-confidence (increased self-efficacy) and goal attainment and lower levels of psychological complaints (depression, anxiety and stress) following the self-management intervention, and (3) whether these effects are maintained after 3, 6 and 12 months, and lastly (4) the cost-consequence of “Partner in Balance” is calculated to estimate the impact of the intervention on resource use, costs, and health outcomes.

## Methods

**Fig. 1:**
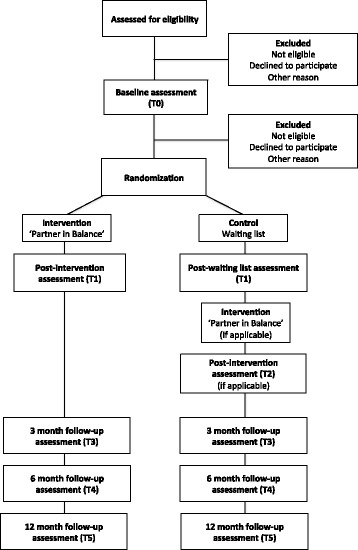
Consolidated Standards of Reporting Trials (CONSORT) flow diagram

The Medical Ethics Committee of the Maastricht University Medical Center + (MUMC+) approved this study (#12-4-059). The study is a randomized waiting-list controlled trial that was designed to maximize acceptability and adherence to the research protocol in the control group and minimize attrition effects. Data will be collected pre intervention and post intervention and at 3-, 6- and 12-month follow-ups. See Fig. 1 for the flow diagram. A full copy of the Standard Protocol Items: Recommendations for Interventional Trials (SPIRIT) checklist for study protocols can be found in Additional file 1.

### Population

#### Recruitment

The study population will consist of family caregivers of community-dwelling people with (very) mild dementia of all subtypes (Clinical Dementia Rating (CDR) score 0.5–1) [30]. No age limit will be applied; however, only adult family caregivers will be solicited. Participants will be recruited from memory clinics (MUMC+, Elkerliek Hospital Helmond, Catharina Hospital Eindhoven) and ambulatory mental health clinics (Virenze-RIAGG Maastricht, MET GGz Roermond) in the south of The Netherlands. Caregivers will be approached by the clinician or therapist who is treating their family member. Prior to participating, caregivers will provide written informed consent.

#### Eligibility

Family caregivers of people with (very) mild dementia of all subtypes (CDR score 0.5–1) who are aged over 18 years, have access to the Internet at home, and have basic skills in the use of computers are eligible to participate in the study. Potential participants who have insufficient cognitive abilities to engage in the online self-management program; are overburdened or have severe health problems, as determined by study staff; or care for people with dementia caused by human immunodeficiency virus (HIV), acquired brain impairment, Down syndrome, chorea associated with Huntington’s disease, or alcohol abuse will be excluded from participation.

### Randomization

Following the baseline assessment, participants will be randomly assigned to either the self-management intervention or the waiting-list control group using a computer program operated by an independent statistician. Block randomization will be conducted to reduce the risk of unbalanced assignment to the experimental and control groups. We will use randomly permuted blocks and several block sizes (4, 6 and 8). The block size and order of allocation will be randomly chosen at the beginning of each block. This reduces the risk of predicting group assignment and keeps research staff blind to the randomization process. An independent research assistant who is unknown to the allocation of the treatment will conduct the post-intervention and follow-up assessments, and will be asked to evaluate the success of blinding, and reasons for possible unmasking, on the Case Record Form.

### Intervention

#### Experimental group

The “Partner in Balance” intervention consists of a face-to-face intake session with a personal coach, an online period, and a face-to-face evaluation session with the personal coach. The basis of the intervention is learning to identify areas of change and creating personal goals. The development and final intervention are described in detail elsewhere [26]. The goal of the intake session is to familiarize participants with the program and to set goals that they wish to accomplish through their participation using the motivational interviewing technique frequently used to identify change objectives and enhance intrinsic motivation to change [31]. Goals and strategies to achieve these goals are individually determined and depend on the participants’ personal problems, motivation, and capabilities. Given that it is not usual for the elderly to reflect on their problems or concerns thematically, a “toolbox” of themes should be developed to aid the discussion of these issues with the participants [32]. Based on their personal needs and areas of interest, participants will select four out of nine modules in the toolbox, and they will be briefed individually to ensure that they understand the online procedure. The module themes are provided in Table 1. The module contents are described elsewhere [28]. Following the intake, participants will complete their chosen modules online during an 8-week period. Two weeks are allocated for each module, but participants will be allowed to complete the modules at their own pace in accordance with the self-management approach [33]. After the online period, participants will meet with their coach face-to-face to discuss their ability to accomplish goals and cope with future difficulties. Participants will continue to have access to their personal page and modules after the intervention period.

**Table 1:** Themes of the available 'Partner in Balance' modules

“Partner in Balance” modules 1. Acceptance 2. Balance in activities 3. Communication with family member and environment 4. Coping with stress 5. Focusing on the positive 6. Insecurities and rumination 7. Self-understanding 8. The changing family member 9. Social relations and support	

#### The personal coach

The personal coach is an experienced professional (psychologist or psychiatric nurse) from one of the participating sites. Coaches will receive a 1-day training on self-management techniques and online help before the start of the intervention. They will receive supervision from an experienced professional in the fields of psychology and self-management to ensure high-quality feedback.

Coaches support participants in choosing modules that fit their situation, help identify feasible goals, offer techniques to achieve goals and provide general constructive feedback on their assignments. Coaches will be matched with the participants assigned to them via a personal login code.

#### Control group

Participants in the control group will be placed on a waiting list for 8 weeks. After they complete the post-test assessment, they will receive the online self-management intervention. They will receive the same pre-test and post-test attention from the research team as the experimental group.

### Procedure

**Fig. 2:**
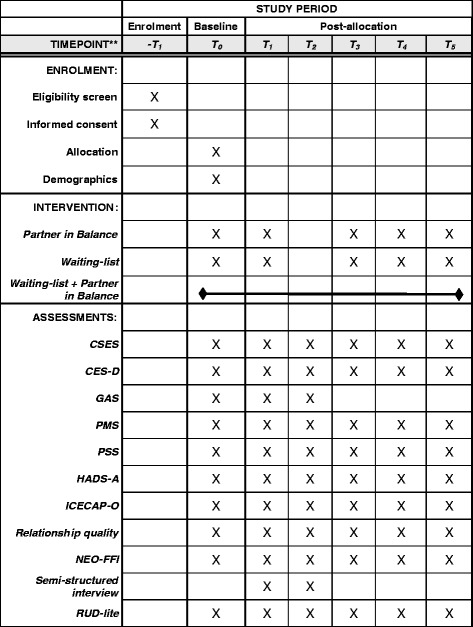
Schedule of enrollment and assessments per condition

Participants will be assessed at five time points: (T_0_) baseline assessment, (T_1_) post-intervention or waiting-list assessment (8 weeks), (T_2_) post-intervention assessment for waiting-list (8 weeks after T_1_), (T_3_) 3-month follow-up, (T_4_) 6-month follow-up, and (T_5_) 12-month follow-up. Figure 2 shows the schedule of enrollment, procedures and mixed-method assessments per condition.

### Questionnaires

#### Primary outcomes

The *Caregiver Self-efficacy Scale (CSES)* [34] is based on a Dutch version of the self-efficacy instrument devised by Lorig et al. [35] and measures care management self-efficacy (four items) and service-use self-efficacy (five items). Item scores range from 1 (not at all certain) to 10 (very certain). Total care management self-efficacy scores range from 4 to 40 and service use self-efficacy from 5 to 50. Good reliability was demonstrated for the CSES in a previous study [28].

The Dutch version of the *Center for Epidemiological Studies Depression Scale (CES-D)* [36] consists of 20 items that rate the frequency of depressive symptoms during the past week, with higher total scores indicating greater depressive symptoms. Scores range from 0 (rarely or none of the time (less than 1 day)) to 3 (most or all of the time (5 to 7 days)). The total score ranges from 0 to 60. Items represent depressed mood, feelings of guilt and worthlessness, feelings of helplessness and hopelessness, psychomotor retardation, loss of appetite, and sleep disturbance. The CES-D is sensitive to changes in caregiver depressive symptoms after intervention [37].

#### Secondary outcomes

*Goal attainment scaling (GAS)* [38] is a measure of treatment-induced change. GAS enables comparisons of individuals’ relative success in achieving goals that are individually determined. Scores range from −2 (much less than expected) to +2 (much better than expected), with 0 indicating that the goal is attained. Raw scores are transformed into *T*-scores [39]. *T*-scores include attainment level and a potential weight assigned to the goal(s). *T*-scores of ≥50 indicate effective goal attainment. GAS has demonstrated reliable monitoring of progress over time [40].

The *Pearlin Mastery Scale (PMS)* [41] measures perceived control, otherwise known as mastery. The scale includes seven items, with scores ranging from 1 (completely agree) to 5 (completely disagree). The sum score (range 0–35) indicates the extent of perceived control or mastery, with higher scores indicating greater perceived control. The psychometric properties of the Dutch version of the PMS have found to be good in previous studies [42].

The *Perceived Stress Scale (PSS)* [43] measures overall appraisals of stress in the past month. Ten items on unpredictability, control and overload are rated on a 5-point scale from 0 (never) to 4 (very often), with higher total scores indicating higher levels of stress. The scale showed good reliability [44].

The *Hospital and Anxiety Depression Scale (HADS-A)* [45] is used to generate scores for generalized anxiety. The anxiety subscale consists of seven items rated from 0 (not at all) to 3 (a great deal of the time). Total scores range from 0 to 21, with higher scores indicating more anxiety. Good reliability was found [46].

The *Investigating Choice Experiments for the Preferences of Older People CAPability measure for Older people (ICECAP-O)* [47] measures five important attributes of quality of life. The value system for the 1024 states defined by the instrument was derived from a survey of older people in England, using a best–worst scaling valuation method. The value system provides a single summary score, anchored at 0 (“no capability”) and 1 (“full capability”), for each state described in terms of the five attributes. The ICECAP-O may have the potential to measure broader outcomes and be more sensitive to differences between intervention and comparators than the EuroQol five dimensions questionnaire (EQ-5D), which is often used in cost-effect evaluations [48].

#### Additional measures

Quality of the relationship is rated with four items of the *University of Southern California Longitudinal Study of Three-Generation Families measures of positive affect* [49]. The items indicate (1) general closeness, (2) communication, (3) similarity of views about life, and (4) degree of getting along. Scores range from 1 (not at all) to 4 (very). The value system for the 1024 states uses a best–worst scaling valuation method, providing a single summary score, anchored at 0 (“no capability”) and 1 (“full capability”). Good reliability was found in a previous study [50].

The *NEO Five-Factor Inventory (NEO-FFI)* [51] measures personality. The 12-item Neuroticism domain will be used to identify individuals who are prone to psychological distress. This domain assesses six traits: anxiety, angry hostility, depression, self-consciousness, impulsiveness and vulnerability [52]. The reliability of the Dutch version of the NEO-FFI is good [51].

#### Semi-structured interview

After the intervention has been completed, a semi-structured interview will be conducted to qualitatively evaluate the effect of the program on participants’ self-efficacy. The interview will take place face-to-face in the caregiver’s home or at the participating institution and will be audiotaped with the participants’ permission. Topics include the application of the intervention in daily life and the intervention’s impact on knowledge about the disease, caregiver functioning, and self-esteem.

#### Process outcomes

Prior to the analysis of effects, the internal and external validity of the intervention will be evaluated using data on sampling quality (recruitment, randomization, and reach) and intervention quality (relevance, feasibility, and performance according to protocol) to provide essential information on the program’s credibility and generalizability [29]. A description of the recruitment and randomization procedure, the informed consent and allocation procedure, and the barriers and facilitators to recruiting caregivers will be provided. Reach will be determined by the proportion of caregivers participating in the study and the number of institutions involved in the intervention. Intervention quality will be determined on two levels: (1) coach, and (2) participant. Data will be collected from the research database, and during focus group interviews with coaches and individual semi-structured interviews with participants post intervention. Coaches will be asked to complete a questionnaire about clearness, relevance, and usability of the intervention. Delivery of the intervention according to protocol will be evaluated with a structured registration form (Additional file 2). For an overview of the specific methods used for each aspect of validity, see Table 2.

**Table 2:** Methods used to assess process of the intervention

Process aspects	Methods used
Sampling quality	Procedure of recruitment, informed consent, and allocation; recruitment barriers and facilitators; reach (proportion participants/institutions)
Intervention quality (rated by coach)	
Protocol deviations; amount and intensity of contact with participant	Registration form (Additional file 2)
Relevance for caregivers and professional coaches; usability in daily practice	5-point rating scale^a^
Advantages of the program; disadvantages/suggestions for improvement; recommendation to other professionals	Open-ended questions
Intervention quality (rated by participant)	
Number of online visits and time spent per module	Online system usage data^b^
Compliance to the program	Ratio number of modules chosen/completed
Clarity of the content; website ease of use; satisfaction with online aspect combined with face-to-face contact; satisfaction with personal coach and feedback; advantages of the program; disadvantages/suggestions for improvement; impact on understanding and knowledge; impact on caregiver self-esteem; recommendation to other caregivers	Semi-structured interview

^a^1 = totally disagree to 5 = totally agree

^b^Online system usage data include frequency of logging in to the system, length of visit (per module feature), and time and date of visit

#### Intervention costs

To provide a comprehensive presentation of the value of “Partner in Balance,” a cost-consequence analysis will be conducted. This is a listing of all the relevant costs and consequences (outcomes) of the intervention. Costs will be based on the Dutch guidelines for cost calculations in health care [53]. Formal and informal resources used by the caregiver and care recipient will be mapped by means of the Resource Utilization in Dementia – shortened version (RUD-lite) [54], which includes information on hospital costs, contacts with the GP or other health care professionals, home care, day care, admissions to a nursing home or home for the elderly, medication and acquisition of goods/aids. Costs are calculated by multiplying the volume of resource use by the cost price per resource unit and include the period from the baseline assessment up to the last follow-up measurement (12 months). Intervention costs include time spent on the intake and evaluation session, e-mail contact, the coach training sessions, materials, and coach supervision. The coaches will register the amount of time spent on the intake, evaluation session and e-mail contact on a structured registration form. All costs are expressed in Euros and are adopted from Hakkaart-van Roijen [53].

### Sample size

We determined our sample size based on previous online intervention studies on caregivers of people with dementia using the CSES as outcome measure, the use of repeated measures, within-between interaction with a mean effect size of 0.2 [55], the following assumptions: alpha 0.05, power 85 % and 25 % loss to follow-up. In accordance with these criteria, we aim to enroll 80 participants (40 participants per group).

### Statistical analyses

#### Quantitative measures

Prior to the analysis, data will be checked for missing values, outliers, and normality. Possible differences between the study groups’ baseline characteristics will be tested with *t* tests for continuous variables and χ^2^ tests for categorical variables. In case of missing data, we intend to test if data is missing completely at random (MCAR) based on a comparison of the baseline characteristics of study completers and participants with missing values. If *p* <0.05 for (one of) the variables in the model, the missing values are non-random. In case of data MCAR, the specification of a missing value or dropout model is not necessary and list-wise deletion will be applied. In case of missing data not at random, we will apply a multiple imputation-based strategy [56].

To examine differences in the outcomes of the intervention and the waiting-list control groups during the intervention period, an analysis of covariance (ANCOVA) will be conducted with post-intervention outcome as the dependent variable, intervention (“Partner in Balance,” waiting-list control group) as the between-subjects variable and the pre-intervention outcome, emotional instability, quality of the relationship, educational level and relationship to the care recipient as covariates. Each outcome will be assessed as a dependent variable. Group differences in the post-intervention outcome adjusted for its baseline value will be examined to test the primary hypothesis. If differences are found, the inter-group effect size will be calculated according to Cohen’s *d*.

To analyze changes in the primary and secondary outcomes during the total study period, the data from the intervention-only group and the waiting-list group receiving the intervention after 8 weeks will be combined using a linear mixed model (LMM). The LMM will estimate fixed effects (regression slopes) for the change during the waiting-list interval (T0–T1 as measured in the waiting-list group only) and fixed effects for change in the intervals during (T1–T2) and after (T2–T3, T3–T4, T4–T5) the intervention period (measured in the waiting-list group after the first 8 weeks and in the intervention group). This will allow us to compare the rate of change in those receiving no intervention (T0–T1) with those receiving the intervention (T1–T5) while accounting for the fact that data are nested in individuals and, therefore, correlated. Intervals will be entered as a categorical variable (five levels) using dummies. Model fit of models with random intercepts (at the participant level) and models with random intercepts and random slopes (at the interval level) will be compared using likelihood ratio tests.

Emotional instability and quality of the relationship will be included as covariates because they are expected to influence the difference between groups. Coach will be introduced into the analysis as a random factor to estimate the variability attributable to the coach. For goal attainment, descriptive statistics will be used to calculate the total number of goals set per domain. Mean goal attainment scaling scores (*T*-scores) will be calculated with a standard formula [38] for each measurement time point.

All analyses will be carried out according to the intention-to-treat principle using IBM SPSS statistics 22.0 for Macintosh. All tests of significance will report mean change and will be two-tailed, with α set at 0.05.

#### Semi-structured questionnaire and process evaluation

The qualitatively obtained data from the semi-structured interview will be transcribed verbatim. Two independent researchers will perform deductive content analysis of the transcribed text using ATLAS.ti. Conceptual labels will be assigned to textual fragments and organized into categories. Categories will be merged into common themes in a consensus meeting. Disagreements will be solved through discussion. The quantitative items scored by coaches will be calculated by means of descriptive statistics.

### Monitoring and participant safety

Monitoring of the recruitment and execution of the study will be conducted by the trial monitoring committee of the MUMC+ (Clinical Trial Center Maastricht). Adverse events (AEs) and serious adverse events (SAEs) are not anticipated but cannot be ignored. If participants drop out, they will be asked if they had experienced an adverse or harmful event during the study period that could be attributed to “Partner in Balance.” Included participants will be asked the same question during the post-intervention assessment and at the 3-, 6- and 12-month follow-ups. All AEs and SAEs will be recorded. SAEs will be reported to the accredited Medical Ethics Committee that approved the protocol. AEs will be followed until they have abated or until a stable situation has been reached. Depending on the event, follow-up may involve additional tests or medical procedures, as indicated, and/or referral to the general physician or a medical specialist. If participants do not agree to this procedure, they cannot participate in the study.

## Discussion

In this paper, we described the design of a randomized waiting-list controlled trial to evaluate the effectiveness and process of a blended self-management program to improve caregiver self-efficacy and psychological well-being. As recommended by the MRC Framework [27], this study was preceded by an iterative development and feasibility study [28]. There are several unique aspects of the current study. To our knowledge, this is the first blended care intervention for early-stage dementia caregivers developed with potential users and tailored to the individual user.

Furthermore, the MRC Framework suggests that implementation should be considered during the first phases of intervention development and evaluation [27]. Implementation is expected to be successful because this intervention was developed with potential professional users and will be evaluated in multiple institutions and with coaches from different backgrounds. The program will be delivered in daily practice and can be integrated in the present care provided. Furthermore, tailoring to individual caregiver needs is expected to increase program effectiveness [17] and facilitate implementation [57]. Additionally, the GAS enables identification of individual benefits that caregivers experience beyond the generalized measures [58]. The process evaluation facilitates our understanding of the quantity and quality of intervention delivered and evaluates the generalizability of the research based on an understanding of the context [59]. The mixed-methods approach enables better understanding of whether and how the intervention works, and facilitates replication of the intervention through greater knowledge of the active component(s) and potential barriers to implementation [60]. Because all the resources used, costs and outcomes are transparently listed in the cost-consequence analysis, decision-makers can select the information that is of most interest to them. Finally, the waiting-list controlled design of the study allows all potentially interested participants to participate in the intervention program. It may increase caregivers’ motivation to participate given that usual care for (very) mild dementia caregivers often either does not include counseling or includes only very infrequent counseling [61].

In conclusion, the results of this study will be a valuable contribution to the growing knowledge on e-health for dementia caregivers. “Partner in Balance” is expected to be effective in increasing caregiver self-efficacy and reducing depressive symptoms in early-stage dementia caregivers. The study will also provide insight into program delivery and program costs related to the program consequences. The results will be used to inform clinicians and researchers of the delivery, costs and effects of “Partner in Balance” as a tool to support dementia caregivers.

### Trial status

Participant recruitment for this study commenced in September 2014, and the study is currently recruiting early-stage dementia caregivers.

## Additional files

Additional file 1: SPIRIT checklist.pdf. (PDF 417 kb) Additional file 2: Registration form coaches “Partner in Balance”.pdf. (PDF 94 kb)

## Abbreviations

CONSORT: Consolidated Standards of Reporting Trials
MRC: Medical Research Council
SPIRIT: Standard Protocol Items: Recommendations for Interventional Trials
