# Supplementary material for: Effectiveness of the blended care self-management program “Partner in Balance” for early-stage dementia caregivers: study protocol for a randomized controlled trial
Source: Trials. 2016 May 4;17:231. doi: 10.1186/s13063-016-1351-z (PMC4855318; doi:10.1186/s13063-016-1351-z)
Supplement: Additional file 1: — SPIRIT checklist.pdf. (PDF 417 kb) [file 13063_2016_1351_MOESM1_ESM.pdf]

Effectiveness of the blended care self-management program 'Partner in Balance' for early-stage dementia caregivers: study protocol for a randomized controlled trial v5 10.09.2015 SPIRIT checklist

**Additional file 1. SPIRIT Checklist**

### 1. *Title*

Effectiveness of the blended care self-management program 'Partner in Balance' for early-stage dementia caregivers: study protocol for a randomized controlled trial

### 2. *Trial registration*

**2a**. Dutch Trial Register: **NTR4748**
http://www.trialregister.nl/trialreg/admin/rctview.asp?TC=4748

**2b**. See Table 1 for full list of trial registration data.

### 3. *Protocol version*

Issue date: 10 September 2015
Protocol Amendment Number: 05
Authors: Lizzy Boots, Marjolein de Vugt, Frans Verhey, Gertrudis Kempen

| | |
|---|---|
| 23-06-2014 | Original |
| 10-09-2014 | Amendment 1: informed consent procedure: contact participants after 1 one week instead of 2 weeks. |
| 23-10-2014 | Amendment 2: New participating center: 'Metggz Roermond' |
| 12-02-2015 | Amendment 3: New participating center: 'Elkerliek hospital Helmond' |
| 29-07-2015 | Amendment 4: flexible intervention period, new follow-up timepoints (6 and 12 months), target audience all family caregivers (not exclusively spousal caregivers) |
| 10-09-2015 | Amendment 5: New participating center: 'Catharina hospital Eindhoven' |

### 4. *Funding*

This study is made possible by the financial support obtained from Alzheimer Nederland (Grant nr WE03-2010-08). All materials developed as part of this study have been funded from this project.

**Table 1. Trial registration data.**

| | |
|---|---|
| Primary registry and trial identifier | NTR4748 |
| Date of registration in primary registry | 20 Aug 2014 |
| Secondary identifying numbers | NL48760.068.14 |
| Funder | Alzheimer Nederland |

Effectiveness of the blended care self-management program 'Partner in Balance' for early-stage dementia caregivers: study protocol for a randomized controlled trial v5 10.09.2015 SPIRIT checklist

| | |
|---|---|
| Primary sponsor | Maastricht University Medical Center<br>Department of Psychiatry and Neuropsychology<br>Attn. Prof. dr. Frans Verhey<br>P.O. Box 616<br>6200 MD Maastricht<br>the Netherlands<br>T +31 (0)43 3874175<br>F +31 (0)43 3875444<br>f.verhey@maastrichtuniversity.nl |
| Contact for public queries | Dr. Marjolein de Vugt<br>Department of Psychiatry and Neuropsychology<br>Maastricht University Medical Center<br>P.O. Box 616<br>6200 MD Maastricht<br>the Netherlands<br>T +31 (0)43 3877445<br>F +31 (0)43 3875444<br>m.devugt@maastrichtuniversity.nl |
| Contact for scientific queries | Dr. Marjolein de Vugt<br>Department of Psychiatry and Neuropsychology<br>Maastricht University Medical Center<br>P.O. Box 616<br>6200 MD Maastricht<br>the Netherlands<br>T +31 (0)43 3877445<br>F +31 (0)43 3875444<br>m.devugt@maastrichtuniversity.nl |
| Public title | Effectiveness of the web-based self-management intervention 'Partner in Balance' for family caregivers of people with early-stage dementia |
| Scientific title | PrepAD Study: Prepare for Alzheimer Dementia. Effectiveness of the web-based self-management intervention 'Partner in Balance' for family caregivers of people with early-stage dementia. |
| Countries of recruitment | The Netherlands |
| Health condition(s) or problem(s) studied | Informal caregivers, prevention, e-health |
| Intervention(s) | Intervention: The blended care self-management program 'Partner in Balance' aimed at learning skills, taking personal needs as a starting point |
| | Control: Waiting-list control |
| Key inclusion & exclusion criteria | Inclusion criteria: |

| | |
|---|---|
| | • Family caregiver of people with Mild Cognitive Impairment (MCI) or mild dementia of all subtypes as described in the Diagnostic and Statistical Manual of Mental Disorders<br>• Caregiver has access to the Internet at home and already masters basic skills in the use of computers.<br>• Written informed consent is obtained. |
| | Exclusion criteria:<br>• Insufficient cognitive abilities to engage in the online self-management program[1]<br>• Caregivers who are overburdened or have severe health problems[1]<br>• Caregivers who's current or near-future situation demands acute, intensive counseling[1]<br>• Caregivers of people with dementia caused by human immunodeficiency virus (HIV), acquired brain impairment, Down syndrome, chorea of Huntington or alcohol abuse.<br>[1] Based on clinical judgment of knowledgeable practitioner, based on his/her experience with the target group). |
| Study type | Single-blind randomized waiting-list controlled trial |
| Date of first enrolment | September 2014 |
| Target sample size | 80 |
| Recruitment status | Pending |
| Primary outcome | Caregiver self-efficacy and depression |
| Secondary outcomes | Goal attainment, mastery, psychological complaints (feelings of anxiety and perceived stress), and quality of life.<br>Possible modifying variables such as caregiver characteristics (quality of the relationship, coping style, neurotic personality) and intervention aspects (coach) on the intervention effect are also evaluated |

***5. Roles and responsibilities***

***5a. Authors' contributions.***

Marjolein de Vugt (MdV) developed the project proposal and obtained funding. Lizzy Boots (LB), MdV, Gertrudis Kempen (GK) and Frans Verhey (FV) designed the study and the materials. LB drafted the manuscript. All authors critically reviewed and approved the final manuscript.

***5b. Name and contact information for the trial sponsor***

Maastricht University Medical Center
Department of Psychiatry and Neuropsychology
Attn. Prof. dr. Frans Verhey
P.O. Box 616
6200 MD Maastricht
The Netherlands
T +31 (0)43 3874175
F +31 (0)43 3875444
f.verhey@maastrichtuniversity.nl

***5c. Role of study sponsor and funders, if any, in study design; collection, management, analysis, and interpretation of data; writing of the report; and the decision to submit the report for publication, including whether they will have ultimate authority over any of these activities***

The funder has no role in the design of this study and will not have any role during its execution, analyses, interpretation of the data, or decision to submit results. The sponsor is responsible for auditing study data and dissemination.

***5d. Composition, roles, and responsibilities of the coordinating center, steering committee, endpoint adjudication committee, data management team, and other individuals or groups overseeing the trial, if applicable***

The trial co-ordination center is Maastricht University Medical Center. The lead investigators are dr. Marjolein de Vugt and professor Verhey. The executive investigator is Lizzy Boots, MSc.
The steering committee will comprise MdV, LB, GK and FV. The trial steering committee will meet every 3 months during trial execution, analysis, and dissemination. The purpose of the trial steering committee will be to ensure that the processes set in place for execution, analysis and presentation of the findings are robust and transparent. A yearly report on trial progress will be made to the funder, which will go out for external peer-review.

Data monitoring team: The Clinical Trial Center Maastricht will oversee the execution of the trial according to protocol. The data management team will visit the coordinating center four times: (1) as the trial is preparing to commence, (2) after trial commencement, (3) around the trial mid-point, (4) after the last assessment of the last participants. During the visits the data monitoring team will ensure accuracy of trial execution and data management. The trial steering committee will also monitor the trial for adverse events (see section 22 – Harms). A report will be made to the steering committee after each meeting.

6. ***Background and rationale***

Effectiveness of the blended care self-management program ‘Partner in Balance’ for early-stage dementia caregivers: study protocol for a randomized controlled trial v5 10.09.2015 SPIRIT checklist

***6a. Background:*** Family caregivers are currently becoming the main source of care for people with dementia [1]. However, dementia caregivers are at risk for depression, anxiety and other health problems [2]. Interventions aimed at reducing the burden of care might reduce dementia care costs in the long term if institutionalization can be postponed [3].

Many face-to-face caregiver support interventions have proven to be effective on caregiver distress (random effect size = 0.3), caregiver knowledge (random effect size: 0.5) [4], and self-efficacy (effect sizes ranging from 0.3 – 0.9) [5]. However, the expected future increase in the number of people with dementia raises concerns about whether care professionals can cope with this demand [6]. E-health interventions could serve as cost-effective alternatives for dementia caregiver support [7-9], both increasing caregivers’ access to support and extending the reach of such support [10-13]. The benefits of e-health are increasingly recognized, and remote support for dementia caregivers is growing [14-16]. Previous e-health studies on dementia caregivers show positive effects on caregiver confidence, stress, depression, and self-efficacy with effect sizes ranging from 0.2-0.3 [17]. Provision of tailored information, a coach and the opportunity to interact with other caregivers shows the most promise [17]. Blending face-to-face guidance with online modules increases caregivers’ connection with the therapist and adherence [18, 19]. However, research on the effects of e-health on dementia caregivers lacks methodological rigor [17], and adverse effects of caregiving are often addressed in the later stages of dementia, when caregivers already feel overburdened [20]. Despite their low levels of life satisfaction and high levels of overload, caregivers do not use services in early stages because they do not feel the need for such services or because they struggle with acceptance due their experience of stigma [21, 20, 22]. Supporting caregivers in an early stage of dementia could prevent high levels of burden and psychological problems in the later stages and delay institutionalization [23-26]. However, early-stage dementia caregiver support can be experienced adversely if the care does not suit the caregiver’s personal situation or the stage of the disease. Negative and stigmatizing information can make it difficult for caregivers to identify with and may hamper acceptance [20]. Learning to positively manage life with dementia instead of managing the dementia itself in a self-management program could facilitate caregivers’ adaptation to their new caregiving role. A focus on enhancing positive, intact experiences that are tailored to the individual caregiver’s situation may be more effective in increasing caregiver self-efficacy and reducing the negative consequences of caregiving at later stages [20].

The current paper presents the design of a randomized waiting-list controlled trial, investigating the effects of the blended care self-management program ‘Partner in Balance’. Alongside the effectiveness study, a process evaluation will be conducted to evaluate the internal and external validity of the program. As recommended by the MRC Framework for design and evaluation of complex intervention [27], the program was based on existing literature [17], theory and user- and professional input [28]. The feasibility and preliminary effectiveness were confirmed in a pilot study. Caregivers reported increased feelings of self-efficacy and goal attainment post-intervention. Feedback from the feasibility study was used to adapt the intervention to increase user-friendliness [28].The next objective is to evaluate the process characteristics, effectiveness and cost-consequence of Partner in Balance.

***6b. Choice of comparators.***

Participants in the control group will be on a waiting list for 8 weeks. After they complete the post-test assessment they will receive the online self-management intervention. They will receive the same pre-test

post-test attention from the research team as the experimental group. the waiting-list controlled design of the study gives all potentially interested participants to opportunity to take part in the intervention program. It may increase their motivation to participate, as usual care for (very) mild dementia caregivers is often no or very low-frequent counseling [29]. Furthermore, this design could increase the motivation of professional caregivers to participate in scientific research.

### *7. Objectives*

Specifically, we aim to investigate (1) internal and external validity of the intervention from sampling quality (recruitment, randomization and reach), and intervention quality (relevance, feasibility, and performance according to protocol) prior to the effect analysis to provide essential information on the program's credibility and generalizability [30], (2) if 'Partner in Balance' is superior compared to the waiting list control group (care as usual) in participant's subjective well-being, with an improved subjective self-confidence (increased self-efficacy) and goal attainment, and lower levels of psychological complaints (depression, anxiety and stress) after participating in the self-management intervention, (3) if the effects are maintained after 3, 6 and 12 months after the intervention. Lastly, (4) cost-consequence of Partner in Balance is provided to estimate the impact of the intervention on lifetime resource use and costs and health outcomes.

### *8. Trial design*

The study involves a randomized waiting-list controlled trial. This design was chosen to maximize acceptability and adherence to the research protocol in the control group and minimize attrition effects. Participants will be randomly assigned to receive either the self-management intervention or the waiting list control group. Data will be collected pre- and post-intervention and at 3, 6 and 12 months follow-ups.

### *9. Study setting*

The trial will be coordinated by Maastricht University Medical Center and four additional centers (memory clinics and ambulatory mental health clinics) are participating:

- Elkerliek Hospital Helmond
- Catharina Hospital Eindhoven
- Virenze-RIAGG Maastricht
- MET ggz Roermond

Caregivers will be recruited in each center, with a total study population of 80 caregivers across the five institutions. Institutions will be selected on their daily interaction with dementia caregivers and their interest in taking part in the study. All data handling will be the responsibility of the coordinating center.

### *10. Eligibility criteria*

**Inclusion criteria**

- Family caregivers of people with (very) mild dementia of all subtypes (Clinical Dementia Rating score 0,5-1)
- >18 years
- Access to the Internet at home and already master basic skills in the use of computers

**Exclusion criteria**

- Insufficient cognitive abilities to engage in the online self-management program
- Overburdened or severe health problems as determined by study staff
- Caregiver for PwD caused by human immunodeficiency virus (HIV), acquired brain impairment, Down syndrome, chorea of Huntington or alcohol abuse

**Assessment of eligibility.**

The study population will consist of family caregivers of community dwelling people with (very) mild dementia of all subtypes (Clinical Dementia Rating score 0,5-1) [31]. No age limit will be applied, however, only adult family caregivers will be solicited. Participants will be recruited at memory clinics (MUMC+, Elkerliek Hospital Helmond, Catharina Hospital Eindhoven) and ambulatory mental health clinics (Virenze-RIAGG Maastricht, MET ggz Roermond) in the south of the Netherlands. Caregivers will be approached by the clinician or therapist who is treating their family member. Before participation, written informed consent should be obtained.

***11. Interventions***

The trial CONSORT diagram is shown in Section 13.

***11a. Interventions for each group.***

*Intervention arm of trial*

The blended-care program 'Partner in Balance' consists of a face-to-face intake session with the personal coach, an online period and a face-to-face evaluation session with the personal coach. Learning to identify areas of change and creating personal goals is the basis for the intervention program. The development and the final intervention are described in detail elsewhere [28].The goal of the intake session is to familiarize participants with the program and set goals they wish to accomplish with their participation, using the motivational interviewing technique frequently used to identify change objectives and enhance intrinsic motivation to change [32]. Goals and strategies to achieve these goals are individually determined and depend on the person's problems, motivation, and capabilities. As it is not usual for elderly to reflect on their problems or concerns thematically, a 'toolbox' of themes should be present to be able to discuss these issues with the participants [33]. Based on their personal needs and areas of interest, participants select an average of four out of nine modules in the toolbox and they are briefed individually to make sure that they understand the procedure of the online self-management program. The available modules and their content is described elsewhere [28]. After the intake, participants will complete their chosen modules during an 8-week online period (2 weeks per module), but adhering to the self-management approach [34], participants are allowed to complete the modules at their own pace. After the online period, participants will discuss with their coach in a face-to-face evaluation session if they feel they can accomplish their goals and if they know how to cope with future difficulties. The personal page and modules will remain accessible for participants after the intervention period.

*The personal coach*

The personal coach is an experienced professional (psychologist or psychiatric nurse) from one of the participating sites. Coaches will receive a one-day training in self-management techniques and online help

before the start of the intervention. They will receive supervision during the course of the intervention period to ensure quality and alignment of the feedback of the coaches according to self-management principle from an experienced professional in psychology and self-management.
Coaches are asked to support participants in choosing modules that fit their personal situation, help participants identify feasible goals, offer techniques to achieve goals and provide participants with general constructive feedback on their assignments. Via a personal login code, coaches will be matched with the participants assigned to them.

***Control arm of the trial.***
Participants in the control group will be on a waiting list for 8 weeks. After they complete the post-test assessment they will receive the online self-management intervention. They will receive the same pre-test post-test attention from the research team as the experimental group.

***11b. Discontinuing or modifying the intervention.***
Participants will be able to leave the trial, if they wish to, at any time and for any reason. An end of trial form will be completed for all trial members, detailing the reason for leaving the trial e.g. choosing to leave; illness; death; loss to follow-up. This is not a medical or pharmaceutical intervention, so we will not modify the delivery of the intervention.

***11c. Improving adherence.***
Personal coaches can monitor the use of the online program remotely for the participants assigned to them, e.g. how often they access the systems; and what modules they visit. Coaches contact participants if no activity is monitored after 2 weeks, to offer their support with the online system. The waiting-list controlled design was chosen to maximize adherence to the research protocol in the control group.

***11d. Concomitant care.***
We will not actively prohibit other care interventions during the trial, but will ask about activities that may support or hinder Partner in Balance or the control period. Furthermore, overburdening (actively in need of care) is one of the exclusion criteria, to minimize the probability of concomitant care.

***12. Outcomes***
*Process evaluation*: internal and external validity of the intervention
*Primary outcomes*: self-efficacy and symptoms of depression.
*Secondary outcomes*: goal attainment, mastery, psychological complaints (feelings of anxiety and perceived stress), and quality of life.
*Possible modifying variables*: caregiver characteristics (quality of the relationship, neurotic personality) and intervention aspects (coach).
*Cost-consequence analysis*: description of costs and health outcomes.

**13. Participant timeline**

Effectiveness of the blended care self-management program 'Partner in Balance' for early-stage dementia caregivers: study protocol for a randomized controlled trial v5 10.09.2015 SPIRIT checklist

Participants will be assessed at 5 time points: ($T_0$) baseline assessment, ($T_1$) post-intervention or waiting list assessment (8 weeks), ($T_2$) post-intervention assessment for waiting-list (8 weeks after $T_1$), ($T_3$) 3-month follow-up, ($T_4$) 6-month follow-up, and ($T_5$) 12-month follow-up.

**CONSORT Diagram**

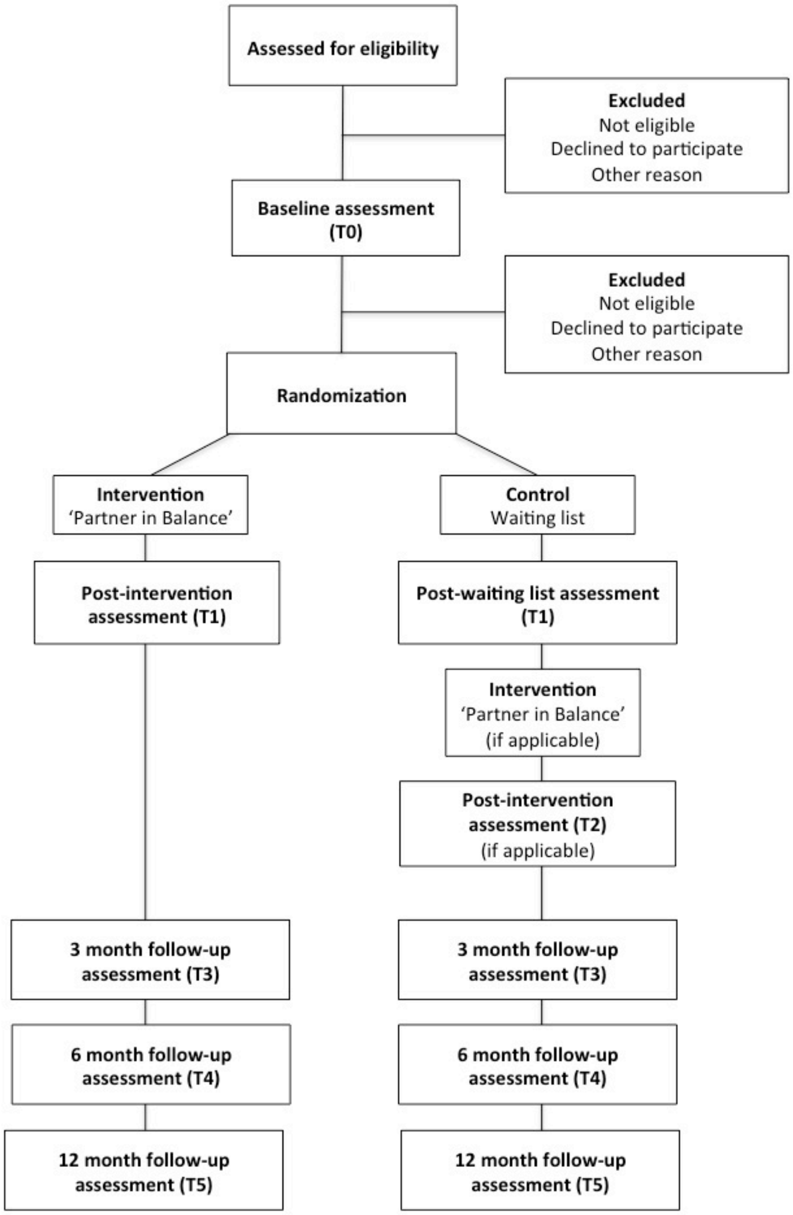


**14. Sample size**

We aim to enroll 80 participants (40 participants per group), based on previous online intervention studies in caregiver of dementia patients with the Caregiver Self-efficacy Scale (CSES) as outcome measure, on the basis of repeated measures, within-between interaction with a mean effect size of 0.2 [35], assuming alpha 0.05, power 85% and 25% loss to follow-up.

**15. Recruitment**

Participants will be recruited at memory clinics (MUMC+, Elkerliek Hospital Helmond, Catharina Hospital Eindhoven) and ambulatory mental health clinics (Virenze-RIAGG Maastricht, MET ggz Roermond) in the south of the Netherlands. Caregivers will be approached by the clinician or therapist who is treating their family member. Before participation, written informed consent should be obtained.

**16. Allocation**

***16a. Sequence generation***

Participants will be randomly assigned to the experimental (self-management intervention) group or the waiting list control group. Randomization will be computer-generated, conducted by an independent statistician and will take place after the baseline assessment. Block randomization will be conducted to reduce the risk of an unbalanced assignment to the experimental and control group. We will use randomly permuted blocks, including several block sizes (4, 6 and 8). The block size and order of allocation will be randomly chosen at the beginning of each block.

***16b. Concealment mechanism***

The randomization sequence will be generated by an independent statistician who will not be involved in the face-to-face recruitment and consenting of participants. Randomly permuted blocks reduce the risk of being able to predict the group assignment and keeps research staff blind to the randomization process. The assessor of the quantitative post-intervention assessments is an independent research assistant who is unknown to the allocation of the treatment.

***16c. Implementation of allocation***

An independent statistician will generate the allocation sequence. The research team will enroll the participants into the trial. After the research assistant has collected baseline data, the researcher will assign participants to either the intervention or control arm of the trial by accessing the computer-generated randomization schedule.

**17. Blinding**

***17a. Trial blinding***

The trial is single-blinded; participants, coaches, and the researcher conducting qualitative research will know the arm to which participants are allocated, however the research assistant conducting the quantitative outcomes will be blinded to study allocation and will be asked to evaluate success of blinding and reason for possible unmasking on the Case Record Form.

***17b. Emergency unblinding***

Emergency unblinding will be the responsibility of the Principal Investigator. Emergency unblinding will only be permitted if there is evidence of intervention-related deaths, as a result of receiving the online intervention

**18. Data collection methods**

Data will be collected with a mixed-methods approach; intervention process and effectiveness will be evaluated quantitatively and qualitatively. The used measures are:

- The *Caregiver Self-efficacy Scale (CSES)* [36], a 10-item self-report scale based on a Dutch version of the self-efficacy instrument of Lorig et al. [37]
- *Centre for Epidemiological Studies Depression Scale (CES-D)* [34]*,* a-20 items self-report scale rating the frequency of symptoms during the past week.
- *Goal attainment scaling (GAS)* [38] is a measure of treatment-induced change, ranging from -2 (much less than expected) to +2 (much better than expected), with 0 meaning goal attainment. Raw scores are transformed into an individual mean GAS score (T-score) [39]. T-scores of ≥50 indicate effective goal attainment.
- *Pearlin Mastery Scale (PMS)* [40], a 7-item self-report scale rating the extent of perceived control or mastery.
- *Perceived Stress Scale (PSS)* [41], a 10-item self-report scale measuring overall appraisals of stress in the past month.
- *Hospital and Anxiety Depression Scale-Anxiety (HADS-A)* [44]*,* a 7-item self-report scale rating generalized anxiety
- *Investigating Choice Experiments for the Preferences of Older People CAPability measure for Older people (ICECAP-O)* [42], a self-completed instrument to measure five important attributes of quality of life.
- *Quality of the relationship* was measured using 4 self-rating items of the University of Southern California Longitudinal Study of Three-Generation Families measures of positive affect [43].
- The 12-item Neuroticism domain of the *NEO Five Factory Inventory (NEO-FFI)* [44], identifying individuals who are prone to psychological distress, by assessing 6 traits: anxiety, angry hostility, depression, self-consciousness, impulsiveness and vulnerability.
- *Semi-structured interview* after completion of the intervention to qualitatively evaluate the effect of the program on participant self-efficacy. This interview will be audiotaped with the participants' permission. Topics include the application of the intervention in daily life, and intervention impact on knowledge about the disease, caregiver functioning, and self-esteem.
- *Process outcomes* from data on sampling quality (recruitment, randomization, and reach), and intervention quality (relevance, feasibility, and performance according to protocol) prior to the effect analysis to provide essential information on the program's credibility and generalizability [30]. Data will be collected using the research database, and semi-structured interviews with coaches
- *Intervention costs* based on the Dutch guidelines for cost calculations in health care [45]. Formal and informal resources used by the caregiver and care recipient will be mapped by means of The Resource Utilization in Dementia – shortened version (RUD-lite) [46], including hospital costs, contacts with the GP or other health care professionals, home care, day care, admissions to a nursing home or elderly home, medication and acquisition of goods/aids. Costs of the online self-management program include time spent on the intake and evaluation sessions performed by the coaches and the e-mail contact, the costs of the training sessions for the coaches, and materials and supervision of the coaches. The coaches will register the amount of time spent on the intake, evaluation sessions and e-mail contact on a structured registration form.

**Summary schedule of assessments**

| | **STUDY PERIOD** | | | | | | |
|---|---|---|---|---|---|---|---|
| | **Enrolment** | **Baseline** | **Post-allocation** | | | | |
| **TIMEPOINT**** | ***-$T_1$*** | ***$T_0$*** | ***$T_1$*** | ***$T_2$*** | ***$T_3$*** | ***$T_4$*** | ***$T_5$*** |
| **ENROLMENT:** | | | | | | | |
| **Eligibility screen** | X | | | | | | |
| **Informed consent** | X | | | | | | |
| **Allocation** | | X | | | | | |
| **Demographics** | | X | | | | | |
| **INTERVENTION:** | | | | | | | |
| ***Partner in Balance*** | | X | X | | X | X | X |
| ***Waiting-list*** | | X | X | | X | X | X |
| ***Waiting-list + Partner in Balance*** | | ◆— | — | — | — | — | —◆ |
| **ASSESSMENTS:** | | | | | | | |
| ***CSES*** | | X | X | X | X | X | X |
| ***CES-D*** | | X | X | X | X | X | X |
| ***GAS*** | | X | X | X | | | |
| ***PMS*** | | X | X | X | X | X | X |
| ***PSS*** | | X | X | X | X | X | X |
| ***HADS-A*** | | X | X | X | X | X | X |
| ***ICECAP-O*** | | X | X | X | X | X | X |
| ***Relationship quality*** | | X | X | X | X | X | X |
| ***NEO-FFI*** | | X | X | X | X | X | X |
| ***Semi-structured interview*** | | | X | X | | | |
| ***RUD-lite*** | | X | X | X | X | X | X |

### 19. Data management

The data of all subjects will be handled confidentially. A subject identification code list will be used to trace data to an individual subject. The investigator will provide all data of subjects with a number that is not based on the patient initials and birth-date (PAD_0XXX). The key to the code will be safeguarded by the investigator in order to keep the data for a longer period of time. All coded interview data will be audiotaped and transcribed, after which tapes are stocked in a locked area, safeguarded by the investigator. Data will be held securely on the cross-platform database application FileMaker. In FileMaker, data is centralized on a server, which can only be

accessed safely using proven encryption standards. Individual user privileges are only available for the research staff of this trial. Data will be transferred securely to SPSS for analysis. FileMaker will be backed up daily.

**20. Statistical methods**

A detailed statistical analysis plan will be written and approved prior to the analysis. In case of missing data, data will be tested on missing completely at random (MCAR) based on a comparison of the baseline characteristics of study completers and participants with missing values. If p <.05 for (one of) the variables in the model, the missing values are non-random. In case of data MCAR, the specification of a missing value or dropout model is not necessary and list wise deletion will be applied. In case of missing data not at random, we will apply a multiple imputation-based strategy [47]. Analysis of covariance (ANCOVA) will be conducted to evaluate group differences in the post-intervention outcome adjusted for its baseline value, emotional instability, quality of the relationship, educational level, and relationship to the care recipient. To analyze changes in the primary and secondary outcomes during the total study period the data from the intervention-only and the waiting list group receiving the intervention after 8 weeks will be combined using a linear mixed model (LMM). For goal attainment, descriptive statistics will be used to calculate the total number of goals set per domain. Mean goal attainment scaling scores (T-scores) will be calculated with a standard formula [38] for each measurement time point. All analyses will be carried out according to the intention to treat principle, using IBM SPSS statistics 22.0 for Macintosh. All tests of significance report mean change and are two tailed with $\alpha$ set at 0.05.

**21. Monitoring**

Monitoring of the overall trial will be the responsibility of the trial monitoring committee (see Section 5d). Monitoring of the recruitment and execution of the study will be conducted by the trial monitoring committee of the MUMC+ (Clinical Trial Center Maastricht).

**22. Harms**

Serious adverse events (SAEs) are not anticipated during this trial, but unanticipated adverse events are always possible. If participants drop out, they will be asked if they had experienced an adverse or harmful event during the study period that could be attributed to 'Partner in Balance'. Included participants will be asked the same question during the post-intervention assessment and at the 3-, 6- and 12-month follow-ups. All AEs and SAEs will be recorded.

A serious adverse event (SAE) is defined as an untoward occurrence that:
(a) results in death,
(b) is life-threatening,
(c) requires hospitalization or prolongation of existing hospitalization,
(d) results in persistent or significant disability or incapacity,
(e) consists of a congenital anomaly or birth defect, or
(f) is otherwise considered medically significant by the investigator.

SAEs will be reported to the accredited MEC that approved the protocol. AEs will be followed until they have

abated or until a stable situation has been reached. Depending on the event, follow-up may involve additional tests or medical procedures, as indicated, and/or referral to the general physician or a medical specialist. If participants do not agree to this procedure, they cannot participate in the study. Reporting of SAEs will include if, in the opinion of the Principal Investigator, the event was:

- 'related': that is, it resulted from administration of any of the research procedures; and
- 'unexpected': that is, the type of event is not listed in the protocol as an expected occurrence.

Reports of related and unexpected SAEs will be submitted within 15 days of the Principal Investigator becoming aware of the event.

**23. Auditing**

The study may be audited randomly by local audit teams of the Clinical Trial Center Maastricht.

**24. Research ethics approval - Plans for seeking REC/IRB approval**

The research team applied for and obtained the relevant approval from the *Medical Ethics Committee (MEC) Maastricht University Medical Centre* (#12-4-059).

**25. Protocol amendments**

Any change in the study protocol will require an amendment. Any proposed protocol amendments will be initiated by the PI and any required amendment forms will be submitted to the MEC. The Sponsor will determine whether an amendment is non-substantial or substantial and will review all amended documents prior to submission to MEC. Before the amended protocol can be implemented (or sent to other participating sites) favorable opinion/approval must be sought from the original reviewing MEC and Sponsor.

**26. Consent or assent**

The clinician or therapist who is treating their family member will approach potential participants. If the caregiver is interested, the researcher will sent a detailed information letter. After 1 week, the researcher will contact the caregiver to answer any questions concerning the research. Researchers will obtain written consent from participants willing to participate in the trial. Information sheets and consent forms are provided for all participants in the trial. The consent to participate in the study may be revoked at any time without further consequences.

**Confidentiality**

The data of all subjects will be handled confidentially. A subject identification code list will be used to trace data to an individual subject. Only the investigator and the research assistant will be able to access the data. All data will be stored for a maximum of 15 years.

**Declaration of interests**

There are no competing interests.

**27. Access to data**

Data access will be restricted to members of the trial team. All stored data will be anonymized; data will not be stored next to caregiver identifiers. Data will be stored for 15 years.

**28. Ancillary and post-trial care**

This is a prevention trial for early-stage caregivers. Participants will be free to seek whatever care they need or want, as normal, from the institution they are familiar with and from other sources.

**29. Dissemination policy**

Academic dissemination will include a number of formats including peer-reviewed journal papers; reports to organizations working in the field of dementia, and presentations at academic and lay-men conferences. Negative results will also be published.

**30. Informed consent materials**

Informed consent forms and other related documentation will be given to all participants.

**31. Biological specimens**

There will be no biological specimens collected as part of this trial.

**References**

1. Association As. Alzheimer's Disease Facts and Figures. Alzheimers Dement. 2014;10.
2. Brodaty H, Donkin M. Family caregivers of people with dementia. Dialogues Clin Neurosci. 2009;11:217-28.
3. Olazaran J, Reisberg B, Clare L, Cruz I, Pena-Casanova J, Del Ser T et al. Nonpharmacological therapies in Alzheimer's disease: a systematic review of efficacy. Dement Geriatr Cogn Disord. 2010;30:161-78. doi:10.1159/000316119.
4. Brodaty H, Green A, Koschera A. Meta-analysis of psychosocial interventions for caregivers of people with dementia. J Am Geriatr Soc. 2003;51:657-64.
5. Tang WK, Chan CY. Effects of psychosocial interventions on self-efficacy of dementia caregivers: a literature review. Int J Geriatr Psychiatry. 2015. doi:10.1002/gps.4352.
6. Macdonald A, Cooper B. Long-term care and dementia services: an impending crisis. Age Ageing. 2007;36:16-22. doi:10.1093/ageing/afl126.
7. Glueckauf RL, Loomis JS. Alzheimer's Caregiver Support Online: Lessons learned, initial findings and future directions. Neurorehabilitation. 2003;18:135-46.
8. Martin-Carrasco M, Martin MF, Valero CP, Millan PR, Garcia CI, Montalban SR et al. Effectiveness of a psychoeducational intervention program in the reduction of caregiver burden in Alzheimer's disease patients' caregivers. Int J Geriatr Psychiatry. 2009;24:489-99. doi:10.1002/gps.2142.
9. Lewis BA, Williams DM, Neighbors CJ, Jakicic JM, Marcus BH. Cost Analysis of Internet vs. Print Interventions for Physical Activity Promotion. Psychol Sport Exerc. 2010;11:246-9. doi:10.1016/j.psychsport.2009.10.002.
10. Davis Kirsch SE, Lewis FM. Using the World Wide Web in Health-related intervention research. CIN: Computers, Informatics, Nursing. 2004;22:8-18.
11. Harrell KM, Wilkins SS, Connor MK, Chodosh J. Telemedicine and the evaluation of cognitive impairment: the additive value of neuropsychological assessment. J Am Med Dir Assoc. 2014;15:600-6. doi:10.1016/j.jamda.2014.04.015.

12. Serafini JD, Damianakis T, Marziali E. Clinical practice standards and ethical issues applied to a virtual group intervention for spousal caregivers of people with Alzheimer's. Soc Work Health Care. 2007;44:225-43. doi:10.1300/J010v44n03_07.
13. Robinson L, Brittain K, Lindsay S, Jackson D, Olivier P. Keeping In Touch Everyday (KITE) project: developing assistive technologies with people with dementia and their carers to promote independence. Int Psychogeriatr. 2009;21:494-502. doi:10.1017/S1041610209008448.
14. Cheng ST, Lau RW, Mak EP, Ng NS, Lam LC, Fung HH et al. A benefit-finding intervention for family caregivers of persons with Alzheimer disease: study protocol of a randomized controlled trial. Trials. 2012;13:98. doi:10.1186/1745-6215-13-98.
15. Blom MM, Zarit SH, Groot Zwaaftink RB, Cuijpers P, Pot AM. Effectiveness of an Internet intervention for family caregivers of people with dementia: results of a randomized controlled trial. PLoS One. 2015;10:e0116622. doi:10.1371/journal.pone.0116622.
16. Cristancho-Lacroix V, Wrobel J, Cantegreil-Kallen I, Dub T, Rouquette A, Rigaud AS. A web-based psychoeducational program for informal caregivers of patients with Alzheimer's disease: a pilot randomized controlled trial. J Med Internet Res. 2015;17:e117. doi:10.2196/jmir.3717.
17. Boots LM, de Vugt ME, van Knippenberg RJ, Kempen GI, Verhey FR. A systematic review of Internet-based supportive interventions for caregivers of patients with dementia. Int J Geriatr Psychiatry. 2014;29:331-44. doi:10.1002/gps.4016.
18. van der Vaart R, Witting M, Riper H, Kooistra L, Bohlmeijer ET, van Gemert-Pijnen LJEWC. Blending online therapy into regular face-to-face therapy for depression: content, ratio and preconditions according to patients and therapists using a Delphi study. BMC Psychiatry 2014;14. doi:10.1186/s12888-014-0355-z.
19. Wilhelmsen M, Lillevoll K, Risør MB, Høifødt R, Johansen ML, Waterloo K et al. Motivation to persist with internet-based cognitive behavioural treatment using blended care: a qualitative study. BMC Psychiatry. 2013;13. doi:10.1186/1471-244X-13-296.
20. Boots LM, Wolfs CA, Verhey FR, Kempen GI, de Vugt ME. Qualitative study on needs and wishes of early-stage dementia caregivers: the paradox between needing and accepting help. Int Psychogeriatr. 2015;27:927-36. doi:10.1017/S1041610214002804.
21. Brodaty H, Thomson C, Thompson C, Fine M. Why caregivers of people with dementia and memory loss don't use services. Int J Geriatr Psychiatry. 2005;20:537-46. doi:10.1002/gps.1322.
22. MacKenzie J. Stigma and dementia: East European and South Asian family

carers negotiating stigma in the UK. Dementia. 2006;5:233-47.
23. Shim B, Barroso J, Davis LL. A comparative qualitative analysis of stories of spousal caregivers of people with dementia: negative, ambivalent, and positive experiences. Int J Nurs Stud. 2012;49:220-9. doi:10.1016/j.ijnurstu.2011.09.003.
24. Banningh LW, Vernooij-Dassen MJ, Vullings M, Prins JB, Rikkert MG, Kessels RP. Learning to live with a loved one with mild cognitive impairment: effectiveness of a waiting list controlled trial of a group intervention on significant others' sense of competence and well-being. Am J Alzheimers Dis Other Demen. 2013;28:228-38. doi:10.1177/1533317513481093.
25. de Vugt ME, Verhey FR. The impact of early dementia diagnosis and intervention on informal caregivers. Prog Neurobiol. 2013;110:54-62. doi:10.1016/j.pneurobio.2013.04.005.
26. Kjallman Alm A, Hellzen O, Norbergh KG. Experiences of long term ongoing structured support in early stage of dementia - a case study. Int J Older People Nurs. 2013. doi:10.1111/opn.12034.
27. Craig P, Dieppe P, Macintyre S, Michie S, Nazareth I, Petticrew M et al. Developing and evaluating complex interventions: the new Medical Research Council guidance. Bmj. 2008;337:a1655. doi:10.1136/bmj.a1655.
28. Boots L, De vugt M, Withagen H, Kempen G, Verhey Fip. Development and initial evaluation of the web-based self-management intervention ‘Partner in Balance’ for family caregivers of people with early-stage dementia: an exploratory mixed-methods study. JMIR research protocols. 2016.

29. Campbell M, Fitzpatrick R, Haines A, Kinmonth AL, Sandercock P, Spiegelhalter D et al. Framework for design and evaluation of complex interventions to improve health. Bmj. 2000;321:694-6.
30. Leontjevas R, Gerritsen DL, Koopmans RT, Smalbrugge M, Vernooij-Dassen MJ. Process evaluation to explore internal and external validity of the "Act in Case of Depression" care program in nursing homes. J Am Med Dir Assoc. 2012;13:488 e1-8. doi:10.1016/j.jamda.2012.03.006.
31. Morris JC. Clinical dementia rating: a reliable and valid diagnostic and staging measure for dementia of the Alzheimer type. Int Psychogeriatr. 1997;9 Suppl 1:173-6; discussion 7-8.
32. Miller WR, Rollnick S. Motivational interviewing: preparing people for change, second edition edn. New York: The Guilford Press; 2002.
33. Daniels R, van Rossum E, Metzelthin S, Sipers W, Habets H, Hobma S et al. A disability prevention programme for community-dwelling frail older persons. Clin Rehabil. 2011;25:963-74. doi:10.1177/0269215511410728.
34. Lorig KR, Holman H. Self-management education: history, definition, outcomes, and mechanisms. Ann Behav Med. 2003;26:1-7.
35. Beauchamp N, Irvine AB, Seeley J, Johnson B. Worksite-based internet multimedia program for family caregivers of persons with dementia. Gerontologist. 2005;45:793-801.
36. Fortinsky RH, Kercher K, Burant CJ. Measurement and correlates of family caregiver self-efficacy for managing dementia. Aging Ment Health. 2002;6:153-60. doi:10.1080/13607860220126763.
37. Lorig K, Chastain RL, Ung E, Shoor S, Holman HR. Development and evaluation of a scale to measure perceived self-efficacy in people with arthritis. Arthritis Rheum. 1989;32:37-44.
38. Kiresuk TJ, Sherman RE. Goal Attainment Scaling: A General Method for Evaluating Comprehensive Community Mental Health Programs. Community Mental Health Journal. 1968;4:443-53.
39. Kiresuk TJ, Smith A, Cardillo JE. Goal attainment scaling: Applications, theory, and measurement. Hillsdale, NJ, England: Lawrence Erlbaum Associates, Inc.; 1994.
40. Pearlin LI, Schooler C. The structure of coping. J Health Soc Behav. 1978;19:2-21.
41. Cohen S, Kamarck T, Mermelstein R. A global measure of perceived stress. J Health Soc Behav. 1983;24:385-96.
42. Grewal I, Lewis J, Flynn T, Brown J, Bond J, Coast J. Developing attributes for a generic quality of life measure for older people: preferences or capabilities? Soc Sci Med. 2006;62:1891-901. doi:10.1016/j.socscimed.2005.08.023.
43. Lawrence RH, Tennstedt SL, Assmann SF. Quality of the caregiver--care recipient relationship: does it offset negative consequences of caregiving for family caregivers? Psychol Aging. 1998;13:150-8.
44. Hoekstra HA, Ormel J, De Fruyt F. Handleiding bij de NEO Persoonlijkheids Vragenlijsten NEO-PI-R en NEO-FFI [Manual for the NEO Personality Inventories NEO-PI-R and NEO-FFI]. Lisse: Swets & Zeitlinger; 1996.
45. Hakkaart- van Roijen L, Tan SS, Bouwmans CAM. Handleiding voor kostenonderzoek. Methoden en standaard kostprijzen voor economische evaluaties in de gezondheidszorg. Geactualiseerde versie 2010 [Dutch manual for costing in economic evaluations]. Diemen: College voor zorgverzekeringen (CVZ); 2011.
46. Wimo A, Winblad B, Stoffler A, Wirth Y, Mobius HJ. Resource utilisation and cost analysis of memantine in patients with moderate to severe Alzheimer's disease. Pharmacoeconomics. 2003;21:327-40.
47. Garson GD. Missing values analysis and data imputation. North Carolina State University: Statistical Associates Publishers; 2015.
