# Supplementary material for: Effectiveness of the blended care self-management program “Partner in Balance” for early-stage dementia caregivers: study protocol for a randomized controlled trial
Source: Trials. 2016 May 4;17:231. doi: 10.1186/s13063-016-1351-z (PMC4855318; doi:10.1186/s13063-016-1351-z)
Supplement: Additional file 2: — Registration form coaches “Partner in Balance”.pdf. (PDF 94 kb) [file 13063_2016_1351_MOESM2_ESM.pdf]

Additional file 2. Registration form coaches Partner in Balance

Participant number: ________

**Action undertaken**	**Date**	**Time spent**	**(If applicable) On whose initiative?**	**Topics discussed**	**Other remarks**
Preparation<br>- Attending coach training<br>- Reading protocol<br>- Attending coach intervision meetings					
Intake session<br>- Getting acquainted<br>- Needs assessment<br>- Setting GAS goals<br>- Choosing modules + adding modules to account<br>- Explaining online procedure<br>- Contact moment agreements<br>- Providing login information					
Online period<br>- Responding to online assignments (email)<br>- Other online help (support requests, reminders)<br>- Telephone contacts (if applicable)					
Evaluation session<br>- General evaluation of modules and experience with the program<br>- Evaluating GAS goals<br>- Identify need for additional modules or types of aid					
